# Assessing the Lagged Regional Economic Output Gaps After the 2015 South India Flood

**DOI:** 10.1111/risa.70365

**Published:** 2026-09-28

**Authors:** Kehan He, Priti Parikh, Zhifu Mi

**Affiliations:** ^1^ Institute for Climate and Carbon Neutrality The University of Hong Kong Hong Kong China; ^2^ The Bartlett School of Sustainable Construction University College London London UK

**Keywords:** disaster footprint, input–output analysis, sequential interindustry model

## Abstract

Natural hazards can adversely impact society, environment, and human well‐being. The development of mitigation strategies is typically based on estimates of economic losses, but such estimates are difficult when high‐resolution post‐disaster data are scarce. Models such as the input–output model have traditionally served as tools for disaster footprint analysis, although their reliance on equilibrium assumptions and limited treatment of production‐capacity adjustment can constrain their application to short‐run natural‐hazard impacts. This study applies a dynamic sequential interindustry model (DSIM), combining recent developments in dynamic input–output analysis with regression‐based coefficient estimation, to examine conditional temporal output gaps after the 2015 South India Flood. Using monthly industrial production indicators and a two‐region aggregation of the 2015 Indian MRIO table, we investigate aggregate economic propagation between Tamil Nadu and the broader Indian economy. The results should be interpreted as model‐implied, conditional scenario estimates rather than independently validated measurements of the full economic cost of the flood. Under these assumptions, the estimated national output gap is approximately 1.6 times the highest available direct‐damage estimate, suggesting that aggregate interregional spillovers may be policy‐relevant even when only limited post‐disaster data are available.

## 1 Introduction

Throughout history, human societies have always faced severe natural hazards leading to substantial losses in both assets and human lives (Klomp and Valckx 2014; Panwar and Sen 2020). Numerous studies have analyzed the effects of these disasters. India, being one of the largest emerging economies and oldest civilization globally, encounters significant hurdles due to natural hazards. The cost of disastrous natural events is further worsened by the recent changing climate patterns worldwide (Cho et al. 2016). For instance, the devastating 2015 South India Flood serves as a stark illustration of these challenges (van Oldenborgh et al. 2016), being among the most severe flood disasters in recent times.

In November 2015, devastating rainfall battered the Southern India states of Tamil Nadu (TN) and Andhra Pradesh. Most heavy damage was suffered by Chennai, the capital of TN Pradesh; around 60% of Chennai was inundated (Johnsy and Schirinzi 2019). The 2015 South India Flood stands as one of the most devastating natural hazards events to have struck India in recent memory, with its catastrophic consequences arising from an interplay between various natural and human factors. On one hand, the higher‐than‐normal precipitation levels can be attributed to the warming global climate, which in turn intensified the El Nino effect (Dhana Lakshmi and Satyanarayana 2019). Geographically speaking, the coastal regions of Southeast India, including cities like Chennai and its surrounding suburbs, are particularly susceptible to flooding hazards due to their relatively flat terrain (Seenirajan et al. 2017). This vulnerability is further exemplified by the 2004 Indian Ocean tsunami, which caused widespread flooding and resulted in 7793 direct fatalities in the state of TN alone (Network of Emergency Physicians 2007). On the other hand, the human element cannot be disregarded in the exacerbation of the flood's deadly impacts. Prior to the heaviest precipitation, residents of Chennai had been warned about the increased risk of flooding. However, the true severity of the impending flood remained largely unknown and unanticipated. Consequently, negligence emerged as a key human factor contributing to the disastrous outcome (Jobin et al. 2018). Moreover, the rapid expansion and development of Chennai's suburbs have also been implicated in the heightened impact of the 2015 flood (Samraj 2017). The rampant urbanization and lack of proper urban planning led to encroachments on natural drainage systems and traditional lakes (Seenirajan et al. 2017), compromising the region's ability to effectively manage the excessive surface run‐off from high intensity rains.

Chennai, the capital of TN, is one of the eight “metropolitans” with a population of 9.7 million (Tandon 2022). TN is the second largest state in India in terms of its GDP, boasting extensive supply chains that stretch across numerous other regions throughout the country (Huang et al. 2021). Hence, as one of India's most significant economic centers, the extensive damages inflicted upon Chennai and thus, TN as a whole, by the 2015 flood reverberated throughout the entire nation, causing widespread disruption (Rajan and Sridharan 2016). For example, the floods led to shut down of the airport in Chennai and disruption to rail network. Now, there has been extensive work put into a plan to recover from the disaster and mitigate future risks disaster (Mariaselvam and Gopichandran 2016; Bremner 2020). As other study has revealed, the management of flood mitigation is an interplay of economics and politics (Parida 2020). The TN state government has estimated the direct financial loss resulting from the flood to be approximately Rs. 8481 crore (The Economic Times 2015) (1 crore = 10 million INR; at 2015 average exchange rate, 1 INR ≈ $0.0156, so Rs. 8,481 crore ≈ $1.32 billion).

However, quantifying the indirect costs incurred both locally and throughout the rest of India (RoI) presents a significant challenge. Accurately estimating the indirect losses from such disastrous events has proven to be a difficult task in numerous studies due to the complex nature of the interconnected economic systems and the far‐reaching consequences of such calamities. To assess the disaster footprint, some economists have used variations of the input–output (IO) model in the past. The IO Model was used in the 1930s to reveal the interlinkages between economic sectors and regions (Leontief 1936). Drawing from extensive surveys and governmental accounting data, the IO Model bridges the gap between micro‐level production and macro‐level economic flows to facilitate policy decisions (Tan et al. 2019; Miller and Blair 2009). Later, economists developed the environmentally extended IO model to address the nexus between economy and energy (Liang et al. 2010), resource (Nakamura and Nakajima 2005), and even health (Zhang et al. 2017). Multi‐regional IO (MRIO) model are used to study trade activities and supply chains among regions (Albino et al. 2002; Wang et al. 2020). IO price model factors in price formation mechanism to discuss fiscal and monetary policies concerning inflations (Przybyliński and Gorzałczyński 2022), exchange rates (Duan et al. 2020), subsidies (Harun et al. 2018), and more. There have been efforts towards improving IO model towards time domain analysis and enable predictions for economic performances (Barker and Santos 2010; Malik et al. 2014; Mendoza‐Tinoco et al. 2017; Yu et al. 2013; Avelino and Hewings 2019; Avelino 2017). Leontief introduced the dynamic IO model (Leontief 1953, 1970), to explain the interaction between capital investment and production efficiencies on a chronological basis (Duchin and Szyld 1985).

In addition, a number of other analysis tools built on the idea of chronological impact in IO model, such as dynamic inoperability (DIIM), supply bottlenecking (ARIO), and hybridization with computational general equilibrium (CGE) model, to analyze the impact of disasters in the long term (Mendoza‐Tinoco et al. 2017; Zeng et al. 2019; Zeng and Guan 2020; Guan et al. 2020). Such developments normally include non‐linear characteristics, assuming a final steady state will be reached given a long‐term general equilibrium. Moving away from the general equilibrium assumption, Romanoff and Levine (1977) propose the Sequential Interindustry Model (SIM) as an innovative approach to include the time domain analysis into the IO model in a culminative linear interaction. Okuyama et al. (2000, 2004) use the SIM to assess the economic impact of natural hazards in Japan by using quarterly disaggregated hypothetical data.

On the other hand, to introduce the influence of capital building on production capacity, dynamic IO model has been proposed by Leontief (1953, 1970). It models the evolution of the economy and the effects of time‐dependent policies or events (Doraszelski and Pakes 2007). Although the dynamic IO model has been criticized by other researchers for a number of reasons, such as omission of production factor constraints and inability to account for labor inputs (Kurz and Salvadori 2000), some researchers are still working towards the perfection and application of the dynamic IO model (Aulin‐Ahmavaara 2000; Rocco 2019), thus providing a valuable and easier tool for understanding the evolution of economic structures, the interdependence of industries, and the impact of policies and shocks over time. In applied studies, economists have implemented the dynamic IO model to evaluate longer term economic growth scenarios (Cruz et al. 2009). The role of capital investment and utilization in the production process can also be assessed with dynamic IO model (Blair and Miller 2022). In more recent application of dynamic IO model, Ryaboshlyk (2006) proposed disaggregating old and new technologies in the time dimension to model technological change in a time span less than a year, better simulating the effects of technological impulses and their diffusion throughout the economy in discrete time steps.

Based on the methodological integration proposed by He et al. (2024) for linking econometric regression and SIM, this study combines stock‐capacity accumulation and temporal interaction among economic regions to apply a dynamic SIM (DSIM) to the assessment of indirect economic losses over time. The contribution is not the claim that DSIM is a wholly new class of disaster model. Rather, the contribution is a constrained empirical application that asks whether a temporally explicit IO framework can extract useful information about aggregate regional spillovers in a setting where high‐resolution post‐disaster data are scarce. Using the 2015 monthly industrial production index and manufacturing index of Indian regions and the 2015 Indian MRIO table, observations on output levels and final demand are simulated and used to solve for the chronological production and investment coefficients of TN and the RoI. This study employs a multi‐regional input‐output (MRIO) framework using the 2015 Indian MRIO table, with Tamil Nadu (TN) and the RoI as the two regions, to assess indirect economic losses that propagate across regions.

Under a business‐as‐usual (BAU) scenario, this research analyses model‐implied output gaps induced in not only TN but also the RoI. In comparison to the available estimates of loss due to the 2015 South India flood, this study estimates conditional direct and indirect output gaps across India that are higher than current direct‐damage estimates. Through this research, aggregate insights into the possible cascading effects of the disaster are obtained to inform policy discussion related to disaster recovery, urban planning, and risk management. Furthermore, the evaluation of indirect losses can shed light on broader implications of natural hazards for the insurance industry, provided the estimates are interpreted as scenario‐based output gaps rather than direct measures of insured asset loss. By incorporating these findings into future planning and policy‐making efforts, authorities can improve their resilience to the adverse effects of such disasters and emerge better prepared to face the challenges posed by an increasingly volatile global climate.

## 2 Data

### 2.1 Data Processing

In stark contrast to existing IO modeling tools used for disaster footprint analysis, which typically model impacts based on a hypothetical inoperability ratio, this study adopts a reverse approach. This research aims to investigate the disaster footprint of the 2015 South India flood by employing a regression analysis of historical economic data, as explained in the previous section. By calculating the chronological production and investment coefficients, the DSIM constructs a “what‐if” scenario that envisions the economic landscape had the disaster not occurred. Consequently, an estimation for local and national outputs unaffected by the shock can be obtained by adding back the direct damage to TN's final demand. We map the official estimate of direct damage to a reduction in TN's final demand for the purpose of the counterfactual. This treats the direct economic impact of the disaster as a demand‐side shock in both final consumption and investment. Under the DSIM model described by Equation (2), the recovery in output will be simulated by both investments to restore production capacity and consumption demand rebound. This counterfactual input should be understood as a monetary shock proxy rather than a literal claim that physical asset damage is equivalent to final consumption loss. It is used because the DSIM requires a monetary perturbation to generate a counterfactual output path, while the official direct damage estimate is the most authoritative event‐specific shock magnitude available for this flood. The aggregate model does not separately identify capacity destruction, labor disruption, price effects, inventory losses, or disaster‐induced changes in A(t) and B(t). Comparing the differences in total and final outputs between the modeled and actual scenarios will therefore reveal conditional, model‐implied chronological output gaps associated with the disaster event, rather than a complete accounting of every physical and market mechanism. The DSIM also addresses some drawbacks of conventional disaster footprint analysis identified earlier. As an enhancement to IO‐based disaster footprint analysis tools, DSIM incorporates the efficiency of investment into its modeling, offering an additional dimension for consideration when making policy decisions regarding recovery strategies. Furthermore, since the DSIM does not assume an equilibrium state at each time step like other IO and CGE models do, it circumvents inaccuracies that may arise when modeling disequilibrium settings over short periods. By embracing this approach, the DSIM analysis carried out in this research can provide a transparent scenario‐based understanding of the possible economic consequences of the 2015 South India flood. This deeper insight can help policymakers discuss recovery strategies and allocate resources to mitigate the far‐reaching effects of such catastrophes on economies and societies in the future.

In order to perform a regression analysis on the relationships among regions and sectors within a MRIO model, a series of observations on final demands and total outputs is required. However, obtaining accurate measurements of these two indicators for shorter statistical periods can be challenging due to the high costs associated with data collection and processing. To investigate the economic symbiosis among Indian regions and subsequently reveal the impact induced by the disastrous event, an approximation of the final demand and total output for TN and the RoI is necessary. In this research, we utilize the monthly Index of Industrial Production (IIP) for manufacturing as an estimation of the intermediate production levels, while considering the total IIP as a representation of the total output levels for both TN (Evaluation and Applied Research Department 2020) and RoI (OECD 2022) during January 2015 and March 2016. These indices are subsequently employed to disaggregate the corresponding quantities from the 2015 India MRIO table.

### 2.2 Data Limitation

Regrettably, with only 15 monthly observations available over a 15‐month period, the data points provided are insufficient for an analysis for longer period. To effectively conduct a regression algorithm in a modeling system with *n* unknowns, it is necessary to have more than *n* observations on other parameters, such as final demands and total outputs in this case. Furthermore, given that items in IO tables are recorded in monetary units, outputs from different sectors are assumed to be interchangeable, with no distinctions made between them. This assumption implies that a decrease in output in one sector will result in an identical decrease in total outputs. In reality, however, a reduced amount of intermediate input in one sector could render inputs from another sector redundant in the production of the final product. This discrepancy between the model's assumptions and the actual complexities of economic systems highlights the limitations of the current modeling approaches. Consequently, certain compromises must be made, such as aggregating the detailed economic sectors in the 2015 Indian MRIO table compiled by the CEADs database (Huang et al. 2021) into a single region for both TN and RoI.

This simplification results in a 2‐by‐2 dimensional MRIO matrix. By measuring the economic outputs of TN and RoI in identical monetary units, we assume that regional outputs are comparable in value terms for the purpose of aggregation. The aggregation should be understood as a choice to estimate aggregate monetary interregional propagation, not as an assumption that different sectoral goods are physically interchangeable. The model preserves information about aggregate value flows between TN and RoI, but it necessarily loses information about sector‐specific bottlenecks, substitution constraints, and differences among individual RoI states. Utilizing the algorithm developed in Equation (S5), it becomes feasible to ascertain the production and investment coefficients for TN and RoI. To ensure the validity of the model, constraints and estimations are imposed on the regression process. Given that the time resolution in this study is restricted to a monthly basis, it is assumed that this duration provides economic agents with adequate time to respond to any market signals. Consequently, the layers of production and investment coefficients are set at 2, indicating that the time delay for the Indian economy to react to any market signal concludes in the second month.

Hence, this model features two 2‐by‐2‐by‐2 tensors, $A_{\left(t\right)}$ and $B_{\left(t\right)}$, with the some diagonal entries set to 0s (Table 1). The number of free parameters thus falls to 8, which is feasible to identify with 15 monthly observations. However, even a modest increase in granularity quickly becomes data‐prohibitive. For example, adding one sectoral split (two sectors) expands *A*_(_ *_t_*_)_ and *B*_(_ *_t_*_)_ to 4‐by‐4‐by‐2 tensors (64 elements). Even under analogous exclusion restrictions to set inter‐regional flows at *t* = 1 and domestic flows at *t* = 2 to zero, the parameter count remains 32, implying roughly 36 monthly observations (about three years) for stable estimation. This requirement conflicts with a key limitation of our data: the available observation window is only one year, within which we assume $A_{\left(t\right)}$ and $B_{\left(t\right)}$ are time‐invariant. Extending the sample to gain degrees of freedom would likely violate this assumption, as input–output coefficients may drift over time. In principle, higher‐frequency observations (e.g., weekly or daily) could increase effective sample size without lengthening the calendar window. However, the consistent, high‐frequency indicators needed to support such estimation are not yet available in major databases or the existing literature.

**TABLE 1:** Simulated result of *A*_(_ *_t_*_)_ and *B*_(_ *_t_*_)_ for the economic activity interactions of Tamil Nadu and Rest of India in the year of 2015.

	$A_{\left(1\right)}$		$A_{\left(2\right)}$	
	Tamil Nadu	Rest of India	Tamil Nadu	Rest of India
Tamil Nadu	0.2467	0	0	0.0152
Rest of India	0	0.4459	0.2040	0

	$B_{\left(1\right)}$		$B_{\left(2\right)}$	
	Tamil Nadu	Rest of India	Tamil Nadu	Rest of India
Tamil Nadu	0.0100	0	0	0.0158
Rest of India	0	0.0911	0.5770	0

## 3 Methods

### 3.1 Dynamic SIM

As a methodological innovation to the IO model, SIM models how total outputs X, demands Y, and intermediate productions A act in chronological symbioses by splitting them into finite discrete time intervals in time series $X_{\left(t\right)}$, $Y_{\left(t\right)}$, and $A_{\left(t\right)}$, respectively (Romanoff and Levine 1986). Hence, the output $X_{\left(t\right)}$ at time t is determined by two variables: the current demand $Y_{\left(t\right)}$ at time t and total output $X_{\left(t\;-\;1\right)}$ at the previous discrete time of (t−1). The rationale is that what has been produced “today” will signal intermediate production “tomorrow” and propagate into the more distant future recursively. In addition, the modified production coefficients $A_{\left(t\right)}$ in SIM not only reflect the magnitude of intermediate inputs, but also the timing for the intermediate inputs to be fulfilled. For instance, a hundred cars are manufactured and consumed “today,” so that four hundred tires are used in the inventory of car making factories. Thus, receiving the market signal, tire manufacturers will produce four hundred tires “tomorrow” to respond to the consumption signal sent out “today.” In the meantime, tires in the car manufacturing industry may be made quicker than cars’ control chips due to reasons like the nature of engineering process and distances among different industry clusters.

Thus the general form of SIM is given in Equation (1) as follow:

(1) $$X_{\left(t\right)}=Y_{\left(t\right)}+\sum_{i=1}^{l}A_{\left(i\right)}X_{\left(t-i\right)}\;\;\forall \;i<t,\;\mathrm{and}\;y_{t}=0\;\mathrm{if}\;t>m$$

In an early work of Romanoff and Levine (1981), Equation (1) is defined as the responsive production SIM where productions are responses to demand in the past. A further advancement in SIM modeling has been proposed to include anticipatory production of demand from the future. Similarly, there is also proposal to change the Leontief IO model from demand‐driven to supply‐driven Ghosh model, so that outputs are results of supply for productions instead of demand induced productions (de Mesnard 2007). However, the Ghosh model has well‐known limitations regarding the interpretation of supply‐side coefficients and its applicability (Dietzenbacher 1997; Altimiras‐Martin 2024). To avoid unnecessary complications, this research will only work with the general responsive production form of SIM.

In a dynamic IO model, the relationship between production capacity and investment is generalized into the following form.

(2) $$x_{\left(t\right)}=Ax_{\left(t\right)}+B\left[x_{\left(t+1\right)}-x_{\left(t\right)}\right]+y_{\left(t\right)}$$

At a certain time step t, the output level $x_{\left(t\right)}$ becomes the sum of intermediate production $Ax_{\left(t\right)}$, final demand $y_{\left(t\right)}$, and an additional capital stock compilation term $B[x_{\left(t+1\right)}\;-\;x_{\left(t\right)}]$. In the capital stock compilation term, the future output level $x_{\left(t+1\right)}$ is linked to present state. Difference in future production $x_{\left(t+1\right)}$ and present production $x_{\left(t\right)}$ is production capacity expansion, which is multiplied with the investment coefficient B to denote the outputs produced at present state for the purpose of capacity expansion. B in a sense also measures the efficiency in investment activities. If the discrete time form is converted into continuous time form, the dynamic IO model will become the following.

(3) $$x=Ax+B\dot{x}+y$$

Since the first proposal of the dynamic IO model, researchers has put continuous efforts into the methodological advancement. Aulin‐Ahmavaara (1990) has made a comprehensive literature review on the methodological advancement of the dynamic IO model so far. Among all innovations presented in Aulin‐Ahmavaara's review, Johansen (1978) is one of the most significant contribution by providing a specific solution to the differential form (3) of the dynamic IO model under a balanced growth rate scenario. In this case, the increase in demand is exactly matched with the increase in supply.

Since SIM discusses the state of economic structure in a very short period, it is assumed that the economy will not be able to respond by investing in capital equipment or similar means to change its structure, but to change production level in the face of differed market signal. A reasonable improvement to the SIM is to factor in the impact of capital investment on economic output. Hence, in the further improvement of dynamic IO model, Ten Raa (1986) has taken one step further to disaggregate production and investment activities into multiple discrete time steps, thus proposing a multiperiod model. Ten Raa's model has been applied and tested in some practical case studies (Fu and Chen 2009). It is given below in Equation (4):

(4) $$x_{\left(t\right)}=\sum_{s=1}^{l}A_{\left(s\right)}x_{\left(t+1\;-\;s\right)}+\sum_{s=1}^{m}B_{\left(s\right)}\left[x_{\left(t+s\right)}-x_{\left(t+s\;-\;1\right)}\right]+y_{\left(t\right)}$$

It can be easily noted that Ten Raa's model approaches the multiperiod concept in a very similar way of SIM, except that a capital building term of $B\dot{x}$ is included. The capital term in Ten Raa's model, like in the classic dynamic IO model (2), links production capacity expansion and investment through the investment coefficient B. Essentially, the first summation term $\sum_{s=1}^{l}A_{\left(s\right)}x_{\left(t+1\;-\;s\right)}$ in Equation (4) makes $x_{\left(t\right)}$ depend on its historical interaction with the intermediate production matrix $A_{\left(t\right)}$, so that this term adds up all the historical contributions from intermediate productions until time interval l to meet the current output level $x_{\left(t\right)}$. The second summation term $\sum_{s=1}^{m}B_{\left(s\right)}\left[x_{\left(t+s\right)}-x_{\left(t+s\;-\;1\right)}\right]$ adjusts $x_{\left(t\right)}$ using the historical investment in capacity to expand the historical production level from $x_{\left(t\right)}$ to $x_{\left(t+1\right)}$ at past time intervals until m. Adding up the final consumption demand $y_{\left(t\right)}$, we obtains the current output level $x_{\left(t\right)}$.

However, it should be noted that it is controversial to use the dynamic model in analysis demolishing or retirement of production capacity. When production decreases in the next time interval, it may be a result of either reduced or idle capacity. If capacity is only idle, investment would not be necessary to expand production in the future so that the dynamic relationship modelled by B would not be valid. In the event of a disaster event, the idle capacity can be quickly utilized to restore original productivity, thus not having an impact on the productivity and investment coefficients $A_{\left(t\right)}$ and $B_{\left(t\right)}$.

In Equation (4), B is given as a three‐dimension matrix like in SIM model, with its third dimension accounts for the discrete time steps. Hence, the impact of investment on production capacity expansion is modeled to take effect with time lags. If the capital building term is to be included in SIM, the new model will look like Equation (5).

(5) $$x_{\left(t\right)}=\sum_{s=1}^{l}A_{\left(s\right)}x_{\left(t\;-\;s\right)}+\sum_{s=1}^{m}B_{\left(s\right)}\left[x_{\left(t+s\right)}-x_{\left(t+s\;-\;1\right)}\right]+y_{\left(t\right)}$$

Ten Raa's model is also different from SIM as it assumes production for outputs starts instantly with zero lags in time instead of one discrete time step later. For the consistency with past research and more convenient reference for concerned researchers, Ten Raa's model is adopted in this study and given the name DSIM model.

### 3.2 Methodological Innovation

Nonetheless, this study faces a comparable challenge to that encountered in He et al. (2024) when utilizing the regression algorithm outlined in Equation (S5). It is evident that the elements of $A_{\left(t\right)}$ and $B_{\left(t\right)}$ must be constrained to non‐negative values and less than one for practical reasons. However, imposing constraints on the objective variable during a regression algorithm using Moore–Penrose Inverse calculations is not feasible. To address this issue, linear programming techniques, combined with some simplifications, are introduced to the case study.

To begin with, our initial step involved making the most accurate estimation of $A_{\left(t\right)}$ by computing the static production coefficient A using the formula derived from the classical IO model, as illustrated in the Equation (6) below:

(6) A=Z./X

where Z is the intermediate consumption matrix obtained from the 2015 India MRIO table. X is the total output vector for TN and RoI. In this research, it is assumed that domestic consumptions sitting on the diagonal happens within the first time discrete (month). The traded flow of consumptions across regions occurs one time discrete later. The estimation of production delay follows the practice from many other similar studies (Okuyama et al. 2004). Hence, an initial estimation on $A_{\left(t\right)}$ is obtained.

The initial guess of $A_{\left(t\right)}$ is then substituted into the DSIM Model (4) to make a first guess before calculating the chronological investment coefficient $B_{\left(t\right)}$. Manipulation of Equation (4) offers an equation with B on the left to be determined, as shown below in Equation (7).

(7) $$B\;\left[x_{\left(t+1\right)}-x_{\left(t\right)}\right]=x_{\left(t\right)}\;-\sum_{s=1}^{l}A_{\left(s\right)}x_{\left(t+1\;-\;s\right)}-y_{\left(t\right)}$$

The left‐hand side of the equation represents the investment in capital stock accumulation, which has a direct linear relationship with the increase in total outputs up to the subsequent discrete time interval. Examining the right‐hand side of the equation, we can also compute the expenditure on capital stock by subtracting the intermediate input and final consumption from the total output. To achieve the most accurate estimate of the coefficient B, it is essential to minimize the discrepancy between the two sides of the equation. Consequently, an optimization system is established as follows to accomplish this goal.

**Table:** 

Find the	B
that minimizes	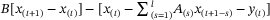
subject to	$0.01<B_{ij}<1.00,\;\mathrm{where}\;b_{ij}\;\mathrm{is}\;\mathrm{any}\;\mathrm{element}\;\mathrm{of}\;B$

In this optimization framework, the boundaries for B are set between 0 and 1 to represent the principle that investments are consistently positive and efficient. This implies that injecting one unit of capital into the economy will yield an output greater than one unit. Upon obtaining an initial static value for B, the same assumption used in $A_{\left(t\right)}$ calculation is employed: domestic consumption occurs within the same time interval, while inter‐regional consumption takes place one time interval later. Consequently, an adjusted $B_{\left(t\right)}$ is derived, serving as the initial estimate of the investment coefficient $B_{\left(t\right)}$.

With the knowledge of the production coefficient *A*_(_ *_t_*_)_ and investment coefficient *B*_(_ *_t_*_)_, it becomes possible to simulate the economic symbiosis between TN and RoI in 2015. If an economic shock event impacts the final demand of any region, the model can simulate the resulting changes—both direct and indirect—across regions in a chronological manner. Specifically, the reduced demand caused by the shock event, such as the direct loss from flooding, is reintroduced to the final demand. The total output changes are then computed in reverse and compared with the actual outcomes to estimate conditional direct and indirect output gaps in the economy. In this case study, the official estimation of Rs. 8500 crores by the TN state government is chosen to represent the direct loss from TN's final demand. This number is selected over other estimates as it is the most authoritative source and best aligns with the trend of TN's monthly growth. Furthermore, to prevent an unrealistic growth rate in this simulation, a cap of 0.6% on the monthly growth rate is imposed by adjusting the 8.0% annual GDP growth rate of India in 2015. This cap is intended to avoid implausible extrapolation, but it also means that the BAU path is not fully independent of observed post‐disaster data. The resulting scenario should therefore be interpreted as a conservative conditional counterfactual rather than an independently validated no‐flood baseline.

## 4 Result Analysis

The production coefficient $A_{\left(t\right)}$ and investment coefficient $B_{\left(t\right)}$ shed light on vital information concerning the chronological interaction between TN and RoI in 2015. Table 1 displays the $A_{\left(t\right)}$ and $B_{\left(t\right)}$ values obtained in this study. Regarding $A_{\left(t\right)}$, a smaller value signifies that fewer resources and inputs are required to produce a unit output for the target region, indicating a higher efficiency in production. Similarly, a smaller value in $B_{\left(t\right)}$ implies that less resources and inputs are needed to increase output by one unit in the subsequent time interval, suggesting a higher efficiency in investment. The indirect losses reported for RoI are presented in aggregate form, as the model treats RoI as a single region. Disaggregation of losses by individual states would require much larger number of observations, which is not feasible for this study. In essence, although sectoral detail is aggregated in the “one sector two region” model, the framework still provides informative measures of interregional economic propagation. Specifically, we interpret the $A_{\left(t\right)}$ and $B_{\left(t\right)}$ coefficients as estimates of cross‐regional multipliers—that is, the total (direct and indirect) increase in output generated across the two regions by a one‐unit increase in final demand, as it triggers intermediate input requirements and induced investment along the supply chain. While this aggregation does not allow us to identify sector‐to‐sector linkages, the resulting multiplier still summarizes the strength of interregional spillovers and is therefore a meaningful quantity for our research question.

In the context of production efficiency, TN's intermediate production exported to RoI (0.0152) represents the most efficient industrial linkage, being 29.3 times more efficient than the domestically produced inputs from RoI itself (0.4459). This occurs despite the exports from TN taking a longer time to reach RoI due to various barriers, such as geographical distance. In contrast, the difference between domestic (0.2467) and imported (0.2040) production efficiencies for TN is relatively small. This reveals TN's role as an economic hub, as its products are more advantageous for RoI compared to those from other regions. A similar comparative advantage is not observed for TN's domestic production over its imports from RoI, further emphasizing the competitiveness of TN's products over imports from RoI in domestic economic activities.

The lower domestic (0.0100) and interregional (0.0158) investment coefficients for TN also demonstrate that its investment efficiency significantly surpasses that of RoI. In contrast, the considerably higher values of domestic (0.0911) and interregional (0.5770) investment coefficients for RoI indicate a clear comparative disadvantage when it comes to investing and stimulating output growth. The model developed in this study estimates that investments from TN to RoI are 36.5 times more efficient, in quantitative terms, than those from RoI to TN. This further reinforces the conclusion that TN serves as India's economic hub, playing a pivotal role in driving economic growth across all regions. This highlights the importance of resilience planning to support sustained development of such economic hubs to ensure growth and ability to withstand climate induced shocks and stresses.

To examine the internal consistency of the developed model, a comparison is made between the simulated outputs for both TN and RoI and the actual outputs observed during the period from April 2015 to April 2016. This comparison is visually presented in Figure 1. Area shaded in red means that the simulated value for output is larger than the actual sample. Area shaded in green means that the simulated value for output is smaller than the actual sample. The number on the horizontal axis means number of months from January 2015, for example, 6 indicates June 2015. The model's production and investment coefficients are configured to have extended influences over a period of two discrete time steps, or months. The time step in which the final demand has a direct impact on the economy has also been taken into consideration. Within this context, the first three data points on the time dimension have not yet been completely affected by the model's mechanisms. Consequently, data points prior to April 2015 are excluded from this analysis. The limited sample size makes formal out‐of‐sample validation difficult. With only 15 monthly observations and 8 free parameters, a conventional train‐test split would leave too few observations either to estimate coefficients or to evaluate prediction error reliably. We therefore use Figure 1 as an in‐sample diagnostic rather than definitive evidence of predictive accuracy. The results of the analysis reveal that the differences from the actual output and the fitted output range from 0.2% to 2.1% for TN and from 0.0% to 2.0% for RoI. These relatively low fitted errors indicate that the constrained DSIM can reconstruct the observed aggregate TN‐RoI output paths within the sample period, but they should not be interpreted as independent validation of forecasting performance. The sign and magnitude constraints imposed on *B*_(_ *_t_*_)_ serve as regularization by preventing economically implausible investment coefficients. Scenario simulations are therefore used as conditional counterfactual exercises based on the chosen shock magnitude, growth cap, and coefficient‐stability assumption.

**FIGURE 1:**
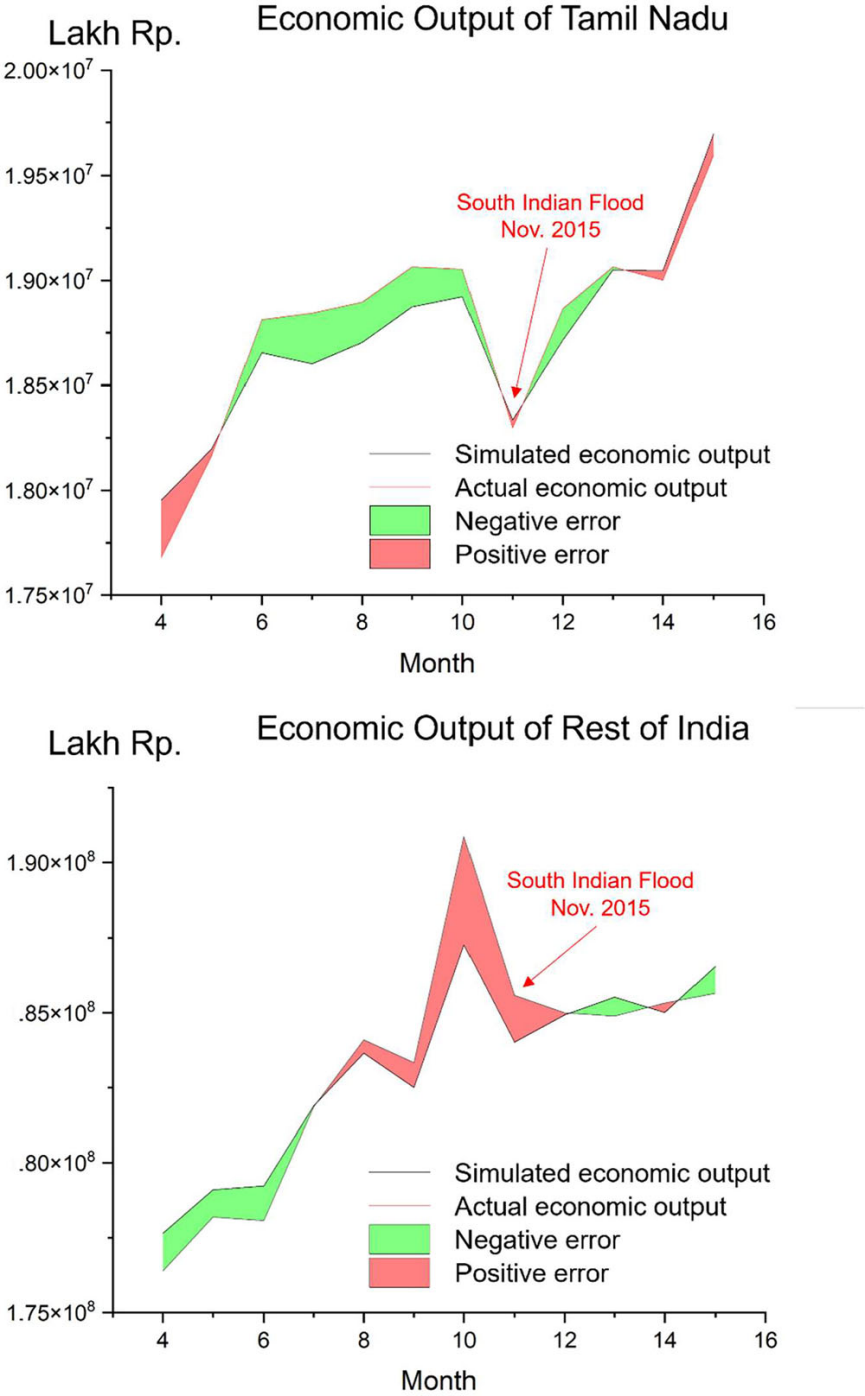
Sensitivity analysis for output simulations of TN and RoI (1 lakh is Rs. 100,000).

To strengthen this in‐sample diagnostic, we further report the per‐row residual root‐mean‐square error of the constrained calibration (TN: approximately 133,300 lakh; RoI: approximately 733,800 lakh) and benchmark the calibrated DSIM against a static specification in which the dynamic capacity‐adjustment term B is set to zero (i.e., a SIM specification). Under the same capped BAU scenario described in the next section, the SIM benchmark yields a TN output gap that is essentially identical to the DSIM result (within 0.2%), while the Rest‐of‐India spillover gap is approximately 18% smaller. This indicates that the dynamic investment channel principally affects the propagation of the shock to the RoI rather than the direct local response in TN, and that the headline DSIM estimate for TN is largely robust to dropping the dynamic term. Per‐row residual diagnostics, fitted‐versus‐observed time series, and the SIM‐versus‐DSIM comparison are reported in Table S1, Table S2, and Figure S1.

To estimate the model‐implied output gap associated with the 2015 South India flood, an unaffected demand for TN is incorporated into the DSIM model to simulate a BAU scenario. The direct damage of Rs. 8500 crore (The Economic Times 2015) is added to the reduced final demand of TN in November 2015, assuming that the flood had not occurred. A monthly overall economic growth rate of 0.6% is presumed, based on the discounted annual growth rate (Evaluation and Applied Research Department 2020). Furthermore, an additional constraint is implemented to restrict the growth of TN's final demand under the hypothetical BAU scenario from surpassing its actual level. The primary objective of this approach is to recreate a conservative conditional scenario in which the economy functions without the direct final‐demand perturbation assigned to the flood. Because the cap uses information from the observed post‐disaster path to avoid implausible extrapolation, the BAU should not be interpreted as a fully independent no‐flood baseline.

To assess how sensitive the model‐implied output gap is to the choice of BAU construction, we further compare three counterfactual paths under the central direct‐damage estimate: (i) the capped BAU described above, (ii) an uncapped BAU obtained by removing the floor at the observed post‐disaster path, and (iii) a pre‐flood‐trend BAU obtained by fitting a log‐linear trend to TN's final demand for January–October 2015 and extrapolating to November 2015–March 2016. The capped and uncapped paths produce identical TN and RoI gaps for this shock magnitude, because the simulated BAU does not dip below observed in any post‐flood month; the cap therefore does not bind in this regime, and the headline result is not driven by the cap itself. The pre‐flood‐trend BAU yields a TN gap that is approximately 85% of the capped value (about Rs. 25,700 crore versus the manuscript's Rs. 30,100 crore), indicating that the magnitude of the model‐implied output gap is moderately sensitive to the underlying BAU construction; the RoI gap is essentially insensitive (within 3%). Full numerical results are reported in Table S3, Table S5, and Figure S1.

The economic outputs of both TN and RoI in time series are illustrated in Figure 2, providing a direct comparison of the flood's impact on not only TN but also other parts of the country over an extended period. Error band widths are estimated based on the percentage error obtained in the previous section. The number on the horizontal axis means number of months from January 2015, for example, 6 indicates June 2015. A noticeable difference is observed in TN's output after November 2015 when comparing the outputs under the BAU scenario to the actual economic output of the flooded scenario (as seen in the left plot of Figure 2). For TN, the actual total economic output nearly aligns with the simulated BAU scenario by April 2016, considering a 2.1% error level based on the in‐sample diagnostic in the previous section. The discrepancy between the two scenarios can be interpreted as a conditional model‐implied output gap generated by the flood scenario. From November 2015 until April 2016, the estimated monthly output gap borne by TN gradually decreased from Rs. 11,500 crore per month to Rs. 1400 crore per month, reflecting the typical characteristics of economic recovery following disastrous events. The cumulative economic output gap for TN during the five months after the flood amounts to Rs. 30,100 crores, which is broadly comparable in order of magnitude with the Rs. 30,000 crore estimation from insurance firms immediately after November 2015 (Vencatesan 2021). We note that insurance typically covers direct asset losses rather than economy‐wide output losses, so this comparison is illustrative rather than a direct like‐for‐like match. This suggests that the model can capture a plausible aggregate recovery trajectory under the stated assumptions, while the numerical estimate should remain interpreted as conditional on the BAU construction and shock proxy.

**FIGURE 2:**
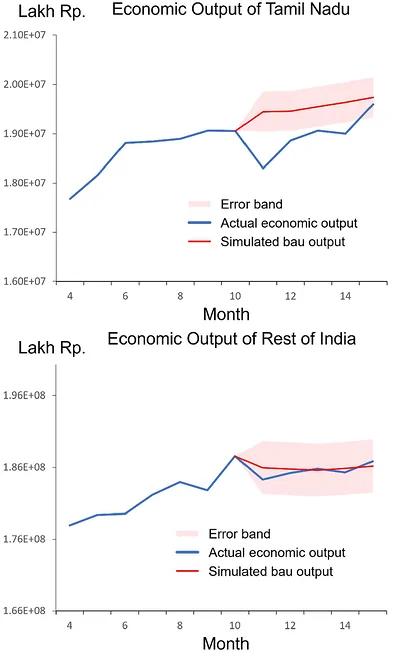
The actual monthly economic outputs of TN and RoI from April 2015 to April 2016 versus simulated BAU scenario using DSIM.

To assess the dependence of these numerical estimates on the chosen direct‐damage figure, we re‐ran the analysis under the lower (Rs. 8500 crore) and higher (Rs. 20,000 crore) rapid assessment estimates. The TN cumulative gap scales monotonically with the shock magnitude, ranging from Rs. 30,100 crore at the manuscript's central estimate to approximately Rs. 73,900 crore at the Rs. 15,000 crore rapid assessment estimate and Rs. 107,700 crore at the Rs. 20,000 crore estimate. The TN gap‐to‐damage ratio is mildly super‐linear (3.54 at the low estimate, 5.38 at the high estimate) because the cap on the BAU path binds in some post‐flood months at low recovery rate but never binds at high recovery rate. The RoI gap scales nearly linearly with the shock (gap‐to‐damage ratio between 2.12 and 2.16). These results confirm that the headline numbers should be read as conditional scenario quantities driven by the chosen monetary shock proxy rather than as independently identified estimates of the full economic cost. Full numerical results are reported in Table S4.

Moreover, the flood's impact extends beyond local regions. The model‐implied economic output gap borne by RoI is estimated to be Rs. 16,400 crores in the month of November 2015 alone by this model, even larger in absolute terms when compared to the gap experienced by TN at the same time. If the accumulated gap from November 2015 to April 2016 is calculated, the total economic output gap of TN (Rs. 30,100 crore) surpasses the total economic output gap of RoI (Rs. 18,300 crore), considering simulated outcomes that are lower than actual outputs. It is worth noting that the simulated BAU economic level of RoI converges with the actual level after December 2015, suggesting that although a spillover impact exists for RoI, it lasts for a relatively shorter period than in TN. In addition, when comparing the sizes of the economies, RoI has undoubtedly suffered less from the 2015 South India flood. The average gap accounts for only about 1.0% of the total economic output of RoI. In contrast, the model estimates an average gap of 3.2% to the economic output of TN, indicating a significantly higher impact compared to RoI. Most importantly, if the estimated economic output gap from RoI is also included in the damage estimation, the conditional national output gap associated with the 2015 South India flood would amount to Rs. 48,400 crores, about 1.6 times of the highest estimation (Rs. 30,000 crore) made by insurance firms. This highlights the ability of the model built in this study to carry out aggregate assessments that consider not only direct losses but also wider ripple effects that disasters may have on interconnected economies. The method used to quantify economic output gaps can serve as a valuable scenario‐analysis tool for both the insurance sector and governmental bodies. This data can aid in developing compensation strategies that more comprehensively alleviate the impacts of disasters by spreading out payments over time. Such an approach can help alleviate financial burdens more comprehensively.

All numbers produced for Figures 1 and 2 are included in Table S1 of the Supporting Information.

## 5 Discussion

In this study, the algorithm developed for DSIM is applied to real data from India in an effort to assess conditional, model‐implied indirect economic output gaps associated with the 2015 South India flood. Under the specific assumptions used here, including the official damage estimate as a monetary shock proxy, a capped BAU path, and stable *A*_(_ *_t_*_)_ and *B*_(_ *_t_*_)_ coefficients, the estimated output gap is larger than available direct‐damage estimates. Rather than a short‐term impact, the DSIM model simulates a prolonged and spilled‐over economic effect on the nation's economy. This study demonstrates that disaster impact assessment can benefit from analytical frameworks capable of capturing temporal dynamics, spatial spillovers, and production capacity constraints simultaneously, while also showing the limits of doing so with sparse monthly data. The substantial spillover effects on RoI, despite its larger economic size, suggest that economic hubs like Chennai can generate cascading impacts throughout national economies. This suggests that disaster risk assessment and mitigation planning should extend beyond directly affected regions to consider interregional economic linkages. The temporal distribution of losses—gradually decreasing from Rs. 11,500 crore per month in November 2015 to Rs. 1400 crore per month by April 2016—provides useful scenario information for designing phased compensation and recovery strategies that align financial support with possible economic recovery trajectories. In practice, this means sequencing spending so that time‐critical measures that restore production capacity and logistics are funded early, and maintaining support long enough to match the projected recovery horizon, rather than ending programs once direct damages are repaired. For RoI, the results indicate that spillovers can be substantial at the onset, implying a need to strengthen resilience beyond the disaster zone. This can be pursued by identifying inputs sourced from flood‐prone areas, diversifying suppliers and transport routes, building operational contingencies, and using buffer inventories where substitution is limited. At the national level, incorporating indirect and growth‐related output gaps into disaster risk reduction planning and into insurance and compensation discussions can improve how resources are mobilized and allocated if considering the result of this research.

However, several limitations of the current study point to important directions for future research. First, the model assumes responsive rather than anticipatory demands, which is appropriate for unanticipated disasters but may limit applicability to policy scenarios where anticipatory behavior occurs (Okuyama 2004). Future work could extend DSIM to incorporate anticipatory demand mechanisms, enabling analysis of both disaster impacts and policy interventions. Second, the current implementation aggregates economic activity into two regions (TN and RoI) due to data availability constraints. This aggregation preserves aggregate monetary flows between the two regions, but it cannot identify sector‐specific bottlenecks, substitution constraints, or heterogeneity among individual RoI states. Sectoral disaggregation would enable identification of which industries drive spillover effects and which sectors are most vulnerable to disaster‐induced disruptions. This would require higher‐resolution IO data and sector‐specific production indices, which may become available as national statistical systems improve. Third, the BAU scenario is a conservative conditional counterfactual rather than a fully independent no‐flood baseline because the growth cap is informed by the observed post‐disaster path. Future work should compare capped BAU results with alternative counterfactuals, such as pre‐flood trend extrapolation or uncapped DSIM scenarios, when the data support such robustness checks. Fourth, the current model maps official direct damage to a final‐demand perturbation as a monetary shock proxy. This does not separately identify capacity destruction, labor disruption, price effects, direct inventory losses, or disaster‐induced changes in *A*_(_ *_t_*_)_ and *B*_(_ *_t_*_)_. Fifth, the model's application is currently limited to India's 2015 flood event. Validation and application to other disaster events—both within India and in other countries—would strengthen confidence in the model's generalizability and help identify whether the 1.6‐fold multiplier found here is consistent across different disaster types, economic structures, and regional contexts. Finally, the current framework focuses on production‐side impacts. Extending the model to incorporate consumption‐side effects, labor market dynamics, and price adjustments would provide a more complete picture of disaster impacts, though this would require additional data and potentially more complex modeling structures.

Finally, the far‐reaching and prolonged regional economic impacts suggested by this conditional analysis highlight the importance of developing robust mitigation and adaptation plans for economic hubs. As climate change increases the frequency and intensity of extreme weather events, ensuring that economic growth is not hampered by shocks and stresses in regional hubs becomes essential for national economic resilience. The DSIM framework provides a quantitative scenario‐analysis tool to inform such planning by revealing both the magnitude and temporal distribution of possible disaster impacts across interconnected regional economies. If higher temporal and sectoral resolution data become available, future work can extend the DSIM framework to capture multi‐regional and multi‐sector dynamic linkages, improving estimates of cascading impacts and enabling more targeted, actionable policy guidance.

## Supporting information




**Supporting File 1**: risa70365‐supp‐0001‐SuppMat.docx

**Supporting File 2**: risa70365‐supp‐0002‐SuppMat.xlsx
