# Supplementary material for: Assessing the Lagged Regional Economic Output Gaps After the 2015 South India Flood
Source: Risk Anal. 2026 Sep 28;46(10):e70365. doi: 10.1111/risa.70365 (PMC13619454; doi:10.1111/risa.70365)
Supplement: Supplementary file 1 [file RISA-46-0-s002.docx]

# Supplementary Information

Figure S1 Robustness analyses for the model-implied output gap

DSIM Algorithm Innovation

Initially, the same recursive algorithm in He et al. (2022) was attempted, in hope to obtain both production coefficient $A\left( t \right)$ and investment coefficient $B\left( t \right)$ in DSIM. However, the recursive algorithm can only work with the case with a single unknown of $A\left( t \right)$. If an extra unknown of $B\left( t \right)$ is to be included in the DSIM model, an alternative algorithm will be needed to solve for the unknown variables and thus conduct an analysis on the operation of the economic.

This section is inspired by the “dynamic inverse” proposed by Steenge and Reyes (2020) to develop an linear algebra algorithm to solve for $A\left( t \right)$ and $B\left( t \right)$ in the DSIM model. In the work of Steenge and Reyes (2020), the capital coefficient $B$ is added to production coefficient $A$, so that the traditional Leontief inverse in IO model is modified to the “dynamic inverse”. Following this practice, DSIM model in is reorganized, so that the final demand $y\left( t \right)$ is expressed as a function of $A\left( t \right)$, $B\left( t \right)$, and $x\left( t \right)$ in ( S.1 ).

| $y_{\left( t \right)}=x_{\left( t \right)}-\sum_{s=1}^{l} A_{(s)}x_{\left( t+s-1 \right)}-\sum_{s=1}^{m} B_{(s)}\left[ x_{\left( t+s \right)}-x_{\left( t+s-1 \right)} \right]$  $y_{\left( t \right)}=x_{\left( t \right)}-\sum_{s=1}^{l} A_{\left( s \right)}x_{\left( t+s-1 \right)}-\sum_{s=1}^{m} B_{\left( s \right)}x_{\left( t+s \right)}+\sum_{s=1}^{m} B_{\left( s \right)}x_{\left( t+s-1 \right)}$  $y_{\left( t \right)}=x_{\left( t \right)}-\sum_{s=1}^{l-1} A_{(s+1)}x_{\left( t-s \right)}-A_{(1)}x_{\left( t \right)}-\sum_{s=1}^{m-1} B_{(s)}x_{\left( t+s \right)}+\sum_{s=1}^{m-1} B_{(s+1)}x\left( t+s \right)-B_{(m)}x_{\left( t+m \right)}+B_{(1)}x_{\left( t \right)}$ $y_{\left( t \right)}=-\sum_{s=1}^{l-1} A_{(s+1)}x_{\left( t-s \right)}+[I-A_{(1)}+B_{(1)}]x_{\left( t \right)}+\sum_{s=1}^{m-1} [B_{(s+1)}-B_{(s)}]x_{\left( t+s \right)}-B_{(m)}x_{\left( t+m \right)}$ | *( S.1 )* |
| --- | --- |

As a linear system, equation ( S.1 ) can easily be converted into matrix form as follow in equation ( S.2 ) to show the final demand of the economy at time $t$.

| $y_{\left( t \right)}=\left[ \begin{matrix} -A_{\left( l \right)} & \cdots& -A_{\left( 2 \right)} & I-A_{\left( 1 \right)}+B_{\left( 1 \right)} & A_{\left( 2 \right)}-B_{\left( 1 \right)} & \cdots& B_{\left( m \right)}-B_{\left( m-1 \right)} & -B_{\left( m \right)} \end{matrix} \right]\times\left[ \begin{matrix} x_{\left( t-l+1 \right)} \\ \vdots\\ x_{\left( t-1 \right)} \\ x_{\left( t \right)} \\ x_{\left( t+1 \right)} \\ \vdots\\ x_{\left( t+m-1 \right)} \\ x_{\left( t+m \right)} \end{matrix} \right]$ | *( S.2 )* |
| --- | --- |

Hence, if the linear system is expanded to show the final demand happened in the economy at all $n$ discrete time steps observed, a matrix multiplication equation ( S.3 ) can be obtained.

| $\left[ \begin{matrix} I-A_{\left( 1 \right)}+B_{\left( 1 \right)} & B_{(2)}-B_{(1)} & \cdots& -B_{(m)} & 0 & & \cdots& & \cdots& & 0 \\ -A_{\left( 2 \right)} & I-A_{\left( 1 \right)}+B_{\left( 1 \right)} & \cdots& B_{(m)}-B_{(m-1)} & -B_{(m)} & & \cdots& & \cdots& & 0 \\ & & & & & \vdots& & & & & \\ -A_{\left( l \right)} & & \cdots& I-A_{\left( 1 \right)}+B_{\left( 1 \right)} & B_{(2)}-B_{(1)} & & \cdots& -B_{(m)} & \cdots& & 0 \\ & & & & & \vdots& & & & & \\ 0 & & \cdots& & & {-A}_{\left( l \right)} & \cdots& I-A_{\left( 1 \right)}+B_{\left( 1 \right)} & \cdots& B_{(m)}-B_{(m-1)} & -B_{(m)} \\ & & & & & \vdots& & & & & \\ 0 & & \cdots& & & & -A_{\left( l \right)} & -A_{\left( l-1 \right)} & \cdots& I-A_{\left( 1 \right)}+B_{\left( 1 \right)} & -B_{(1)} \\ 0 & & \cdots& & & & 0 & -A_{\left( l \right)} & \cdots& -A_{\left( 2 \right)} & I-A_{\left( 1 \right)} \end{matrix} \right]\times\left[ \begin{matrix} x_{\left( 1 \right)} \\ x_{\left( 2 \right)} \\ \vdots\\ x_{\left( n-1 \right)} \\ x_{\left( n \right)} \end{matrix} \right]=\left[ \begin{matrix} y_{\left( 1 \right)} \\ y_{\left( 2 \right)} \\ \vdots\\ y_{\left( n-1 \right)} \\ y_{\left( n \right)} \end{matrix} \right]$ | *( S.3 )* |
| --- | --- |

Let

$$P=\left[ \begin{matrix} I-A_{\left( 1 \right)}+B_{\left( 1 \right)} & B_{(2)}-B_{(1)} & \cdots& -B_{(m)} & 0 & & \cdots& & \cdots& & 0 \\ -A_{\left( 2 \right)} & I-A_{\left( 1 \right)}+B_{\left( 1 \right)} & \cdots& B_{(m)}-B_{(m-1)} & -B_{(m)} & & \cdots& & \cdots& & 0 \\ & & & & & \vdots& & & & & \\ -A_{\left( l \right)} & & \cdots& I-A_{\left( 1 \right)}+B_{\left( 1 \right)} & B_{(2)}-B_{(1)} & & \cdots& -B_{(m)} & \cdots& & 0 \\ & & & & & \vdots& & & & & \\ 0 & & \cdots& & & {-A}_{\left( l \right)} & \cdots& I-A_{\left( 1 \right)}+B_{\left( 1 \right)} & \cdots& B_{(m)}-B_{(m-1)} & -B_{(m)} \\ & & & & & \vdots& & & & & \\ 0 & & \cdots& & & & -A_{\left( l \right)} & -A_{\left( l-1 \right)} & \cdots& I-A_{\left( 1 \right)}+B_{\left( 1 \right)} & -B_{(1)} \\ 0 & & \cdots& & & & 0 & -A_{\left( l \right)} & \cdots& -A_{\left( 2 \right)} & I-A_{\left( 1 \right)} \end{matrix} \right]$$

$$U=\left[ \begin{matrix} x_{\left( 1 \right)} \\ x_{\left( 2 \right)} \\ \vdots\\ x_{\left( n-1 \right)} \\ x_{\left( n \right)} \end{matrix} \right]$$

$$V=\left[ \begin{matrix} y_{\left( 1 \right)} \\ y_{\left( 2 \right)} \\ \vdots\\ y_{\left( n-1 \right)} \\ y_{\left( n \right)} \end{matrix} \right]$$

So that equation above becomes:

$$PU=V$$

Hence to obtain output $U$, multiplying the inverse of $B$ on both sides we get

| $U=P^{-1}V$ | *( S.4 )* |
| --- | --- |

In equation ( S.3 ), the linear system of DSIM is transformed into a matrices multiplication process by setting an unchanging vector of $x_{\left( t \right)}$ and forming a matrix of varying coefficients $A_{\left( t \right)}$and $B_{\left( t \right)}$. Similarly, if the matrix of $A(t)$ and $B(t)$ is held as the unchanging vector and elements in the matrix of $x(t)$ are varied to form a matrix, the identical output of final demand $y(t)$ can be obtained as shown in equation ( S.5 ).

| $\left[ \begin{matrix} -A_{\left( l \right)} & \cdots& -A_{\left( 2 \right)} & I-A_{\left( 1 \right)}+B_{\left( 1 \right)} & A_{\left( 2 \right)}-B_{\left( 1 \right)} & \cdots& B_{\left( m \right)}-B_{\left( m-1 \right)} & -B_{\left( m \right)} \end{matrix} \right]\times\left[ \begin{matrix} 0 & \cdots& 0 & x_{\left( 1 \right)} & x_{\left( 2 \right)} & \cdots& x_{\left( l \right)} \\ & & & \vdots& & & \\ 0 & \cdots& x_{\left( n-l-3 \right)} & x_{\left( n-l-2 \right)} & x_{\left( n-l-1 \right)} & \cdots& x_{\left( n \right)} \\ x_{\left( 1 \right)} & \cdots& x_{\left( n-l-2 \right)} & x_{\left( n-l-1 \right)} & x_{\left( n-l \right)} & \cdots& 0 \\ x_{\left( 2 \right)} & \cdots& x_{\left( n-l-1 \right)} & x_{\left( n-l \right)} & x_{\left( n-l+1 \right)} & \cdots& 0 \\ & & & \vdots& & & \\ x_{\left( m-1 \right)} & \cdots& x_{\left( n-2 \right)} & x_{\left( n-1 \right)} & x_{\left( n \right)} & \cdots& 0 \\ x_{\left( m \right)} & \cdots& x_{\left( n-1 \right)} & x_{\left( n \right)} & 0 & \cdots& 0 \end{matrix} \right]=\left[ \begin{matrix} y_{\left( 1 \right)} & \cdots& y_{\left( n-l-1 \right)} & y_{\left( n-l \right)} & y_{\left( n-l+1 \right)} & \cdots& y_{\left( n \right)} \end{matrix} \right]$ | *( S.5 )* |
| --- | --- |

Again, let ( S.5 ) be $PU=V$, so that a unique solution of $y_{\left( t \right)}$ can be obtained if $U$ is just symmetric. However, in real world situation, observations for $x_{\left( t \right)}$ and $y_{\left( t \right)}$ are normally more than required for a unique solution. Thus matrices $U$ would be asymmetric. The inverse of $U$ is hence impossible to be calculated. We thus introduce the same technique in He et al. (2022) to adopt the Moore-Penrose pseudoinverse of $U$.

| $P=VU^{+}$ | *( S.6 )* |
| --- | --- |

In equation ( S.6 ), $U^{+}$ denotes the Moore-Penrose pseudoinverse matrix of $U$, widely used for linear regression problems (MacAusland, 2014). $U^{+}$ is multiplied on both sides of ( S.6 ) to calculate matrix $P$. Truncating matrix $P$ at $(n-l)$ observations and taking the minus of the $m$ terms of the right-side of $B$, we have a series that looks like:

| $\begin{matrix} A_{\left( 1 \right)}-B_{\left( 1 \right)}-I & B_{\left( 1 \right)}-B_{\left( 2 \right)} & \cdots& B_{\left( m-1 \right)}-B_{\left( m \right)} & B_{\left( m \right)} \end{matrix}$ | ( S.7 ) |
| --- | --- |

So that all investment coefficients $B\left( m \right)$ are solved by the following recursive steps:

| $B_{\left( m \right)}=B_{\left( m \right)}$  $B_{\left( m-1 \right)}=\left[ B_{\left( m-1 \right)}-B_{\left( m \right)} \right]+B_{\left( m \right)}$  $\vdots$  $B_{\left( 1 \right)}=\left[ B_{\left( 1 \right)}-B_{\left( 2 \right)} \right]+B_{\left( 2 \right)}$  $A_{\left( 1 \right)}-I=\left[ A_{\left( 1 \right)}-B_{\left( 1 \right)}-I \right]+B_{\left( 1 \right)}$ | ( S.8 ) |
| --- | --- |

Taking the minus of the $l$ terms of the left-side of $B$, the following series of elements can be obtained which directly gives the series of $A_{\left( t \right)}$.

| $\begin{matrix} A_{\left( l \right)} & \cdots& A_{\left( 2 \right)} & A_{\left( 1 \right)}-B_{\left( 1 \right)}-I \end{matrix}$ | ( S.9 ) |
| --- | --- |

Finally, the term $A_{\left( 1 \right)}$ can be calculated as follow.

| $A_{\left( 1 \right)}=\left[ A_{\left( 1 \right)}-B_{\left( 1 \right)}-I \right]+B_{\left( 1 \right)}+I$ | ( S.10 ) |
| --- | --- |

The proposed algorithm makes it feasible to work out the production and investment coefficients through an ample amount of observations on the outputs and final demands in an economy. Knowing these parameters permits the analysis on the operations of the economy investigated and thus make fair predictions for its performance in the short future.

Reference

HE, K., MI, Z., COFFMAN, D. M. & GUAN, D. 2022. Using a linear regression approach to sequential interindustry model for time-lagged economic impact analysis. *Structural Change and Economic Dynamics,* 62**,** 399-406.

MACAUSLAND, R. 2014. The Moore-Penrose inverse and least squares. *Math 420: Advanced Topics in Linear Algebra***,** 1-10.

STEENGE, A. E. & REYES, R. C. 2020. Return of the capital coefficients matrix. *Economic Systems Research***,** 1-12.
